# Editorial: Aging in the Digital Era

**DOI:** 10.3389/fpsyg.2019.01815

**Published:** 2019-08-07

**Authors:** Carmen Moret-Tatay, Mike Murphy

**Affiliations:** ^1^Departamento de Metodología, Psicología Básica y Psicología Social, Universidad Católica de Valencia, San Vicente Mártir, Valencia, Spain; ^2^School of Applied Psychology, University College Cork, Cork, Ireland

**Keywords:** digitalization, aging, ICT, cognition, technology

Digitization has advanced considerably in the last decade. Devices that a few years ago would have been be considered close to science fiction, not to mention economically unattainable, are currently at our disposal, increasing the means through which we can relate with the world. As expected, the ways in which humans interact and use these technologies have attracted considerable attention in the scientific community, which has led to innovations in technology, as well as theories related to changes in technology adoption according to jurisdiction (Ricardo-Barreto et al.), behavior (Mangen and Velay, 2010; Marco and Tormo-Irun), and in the human brain itself (Greenfield, 2015; Lemus-Zúñiga et al., 2015). These developments take place in the context of a rapidly aging society, and where many disciplines have struggled to address the interaction of technology with the aging process in terms of human development, cognition, social support, and emotional skills (Charness and Boot, 2009; Mangen, 2016; Kuzmičová et al., 2018).

Technology, and more precisely, digitization, makes our lives easier, and it may also be transforming our mental processes. If so, it seems imperative to examine the impact of its use on our cognition. Digitization can obviously lead to certain skills getting worse, but it also leads to an improvement of other abilities (Hu et al., 2017; Wilmer et al., 2017; Uncapher and Wagner, 2018). These improvements may not be measured properly in traditional IQ assessment tools. In this way, recently there has been talk of a slowdown or reversal in several countries of the well-known Flynn effect on the growth of the Intellectual Quotient. Some Researchers predict a change arising from the cognitive demands of the use of new devices, as well as changes in such social domains as family, education and work (Bratsberg and Rogeberg, 2018; Flynn and Shayer, 2018; Oliveira et al.).

One variable of interest in reflecting our cognitive architecture is reading and, more precisely, it seems to be a key skill for full digital citizenship. Therefore, internet-based technologies have been of interest for the scientific community, as they make research in this field more accessible (Dufau et al., 2015; Moret-Tatay et al., 2018). Reading is a complex process that is based on the efficiency of lexical and sub-lexical linguistic skills such as orthographic, phonological, semantic, morphological and syntactic knowledge, to decode and understand a text (Wolf and Stoodley, 2008). This process is even more complex when variables such as the type of reading resource are considered. A survey conducted almost a decade ago (Rideout et al., 2010), suggested that those who read in print texts were less likely to perform multiple tasks than when they read digitally; more precisely, reading digitally appeared to encourage divided attention. Likewise, the printed texts allow readers to see and feel the spatial extent and physical dimensions of the text, and the material of the article provides fixed physical, tactile and spatially temporal keys throughout the reading (Mangen, 2010). Despite the emerging preference for digital texts (Singer and Alexander, 2017) the literature seems not to be conclusive on its impact on cognition. For example, there are certain exceptions in relation to these findings—Schneps et al. (2019) found that groups of readers with dyslexia showed a more effective and compressive reading in digital texts.

Another case where digitization has a clear impact on the cognition is that of human navigation. In real-world situations, such as navigating a city, there may be more than one route to a destination. The more options to consider, the greater demands placed on the brain regions needed to retrieve the network of possible paths and select the optimal route. This process might be affected by the popular use of positioning systems (such as GPS). The spatial navigation carried out by human beings is fundamental to our independence. Therefore, it is not surprising that deficits in spatial navigation can be among the first symptoms of dementia (Gazova et al., 2012). Studies in the field show that this is related to cognitive aspects such as working memory and attention, and that the skill is plastic—a brief navigation training changes a person's brain tissue and improves the way in which the modified tissue communicates with other areas of the brain involved in navigation. According to the literature (Boccia et al., 2014), when navigating through the environment, people can use two basic navigation strategies associated with different internal representations of the space. Self-centered navigation is a body-centered strategy that uses distances and indications to or from individual reference points with respect to the subject's body position. Allocentric navigation is a world-centered strategy that uses information about distances and angles between different locations in the environment, regardless of the subject's position, and is the one that experiences the greatest age-related decline. This issue could be a challenge for futures generations if positioning systems really have an impact in our sense of orientation.

It is also important to understand the barriers to and facilitators of (e.g., technology-related anxiety and self-efficacy) the adoption of emerging technologies, across the lifespan (Moret-Tatay et al., 2019). Some of these factors are of specific interest in the field of aging (Charness et al.; e.g., Rauschnabel et al., 2015), pointed out that the openness trait might predict the use of ICT. According to Bernabé-Valero et al., some other variables may play a crucial role in adoption—in this study, it was found that intellectual humility moderates the adoption process of mobiles and computers.

Finally, technology aims to be accessible for everyone. This is one of the biggest challenges in our society—not only for the older population, but also for other groups, such as people with intellectual disabilities. The needs of this group are related to their condition (Durbin et al., 2017), where psychological or functional impairments needs should be addressed. One opportunity in the field is the use of e-health interventions (Constant et al., 2018). However, this issue becomes even more complex when combined with aging. According to Vázquez et al., after a systematic review in the topic, literature underscores the need to guarantee equity in e-health adoption for this population.

As expected, the ways in which humans interact with and use these technologies has attracted considerable attention in the scientific community, which has led to innovations in digital environments, as well as theories related to changes in behavior and in the human brain itself. The current Research Topic aimed to integrate some of the key empirical evidence that has emerged regarding the association between digitization, society and cognition. Most of the research studies included are concerned with the growing presence of digitization in our culture, domains in which it may enhance cognitive skills, and domains in which the scientific literature is not developed enough to substantiate such claims. Future lines of research should aim to examine evidence relating to both, as well as the consequences in such domains as human intelligence. Even if this last objective is not straightforward, we expect this call can serve as a basis, or at least a resource, for those conducting further research in this area. Understanding the mind, in an integrative way, requires both theory and empirical evidence that provide further knowledge regarding its organization and development, including individual differences, and learning at different phases of development or life span.

## Author Contributions

CM-T and MM conceived of the presented idea and drafted the manuscript.

### Conflict of Interest Statement

The authors declare that the research was conducted in the absence of any commercial or financial relationships that could be construed as a potential conflict of interest.
